# Early identification and brief intervention for risky drinkers: an experience with general practitioners in Florence, Italy

**DOI:** 10.1186/1940-0640-7-S1-A38

**Published:** 2012-10-09

**Authors:** Allaman Allamani, Manuele Falcone

**Affiliations:** 1 Tuscan Regional Agency of Health, Firenze, Italy

## 

Successful implementation of early identification and brief intervention (EIBI) was implemented in Italy from 2005–2007 in the city of Florence and its environs. Twenty-five general practitioners (GPs) identified risky drinkers among their clients and followed them for 12 months. Follow-up included visits to the GP’s office with educational interventions aimed at reducing or stopping drinking. The GPs recorded alcohol consumption, drinking patterns, and conducted blood tests. A computer system was created to collect data centrally. At the end of the study, 2869 clients were enrolled (average daily alcohol consumption, 15.05 g). Three hundred and eight were risky drinkers (10.7%) (42.64 g alcohol on average per day). Of these, 40.6% had at least one abnormal blood test. Although fewer risky drinkers presented for follow-up (n = 126), their decrease of alcohol consumption was relevant at the second visit (37.61 g). Based on this experience, a one-day educational program for GPs on the issue of alcohol and alcohol-related problems was initiated in Florence, starting in 2010. The program focused on health information as well as communication skills, education, rehabilitation, and prevention. Two education groups were implemented in 2010. Forty-six GPs were asked their opinion on BI prior to participating in the educational program via an Italian version of the Brief Intervention Questionnaire. About 90% of participating GPs thought they needed to be better prepared to identify risky drinkers and to implement BI. About 60% found it difficult or somehow difficult to address the issue of drinking with patients. Thirty percent of GPs drank alcoholic beverages daily or nearly daily, and 22.5% smoked cigarettes.

